# Photophysical Characterization of Ru Nanoclusters on Nanostructured TiO_2_ by Time-Resolved Photoluminescence Spectroscopy

**DOI:** 10.1021/acs.jpcc.3c04075

**Published:** 2023-07-15

**Authors:** Kasper Wenderich, Kaijian Zhu, Yibin Bu, Frans D. Tichelaar, Guido Mul, Annemarie Huijser

**Affiliations:** † Photocatalytic Synthesis Group, Faculty of Science and Technology, MESA+ Institute for Nanotechnology, University of Twente, P.O. Box 217, 7500 AE Enschede, The Netherlands; ‡ Nanolab, MESA+ Institute for Nanotechnology, University of Twente, P.O. Box 217, 7500 AE Enschede, The Netherlands; § Kavli Institute of Technology, Quantum Nanoscience, Delft University of Technology, 2628 CJ Delft, The Netherlands

## Abstract

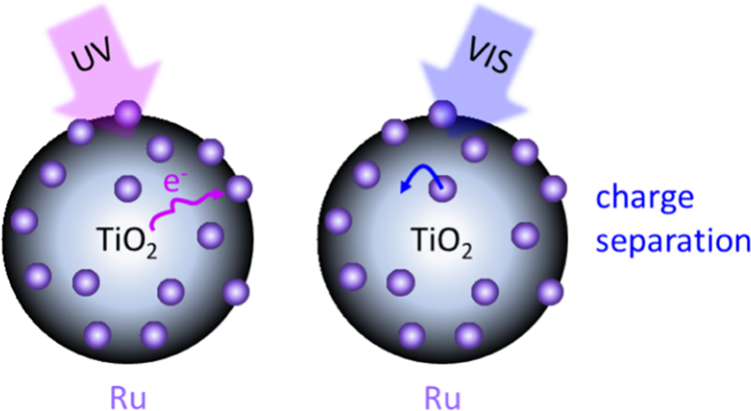

Despite the promising performance of Ru nanoparticles or nanoclusters on nanostructured TiO_2_ in photocatalytic and photothermal reactions, a mechanistic understanding of the photophysics is limited. The aim of this study is to uncover the nature of light-induced processes in Ru/TiO_2_ and the role of UV versus visible excitation by time-resolved photoluminescence (PL) spectroscopy. The PL at a 267 nm excitation is predominantly due to TiO_2_, with a minor contribution of the Ru nanoclusters. Relative to TiO_2_, the PL of Ru/TiO_2_ following a 267 nm excitation is significantly blue-shifted, and the bathochromic shift with time is smaller. We show by global analysis of the spectrotemporal PL behavior that for both TiO_2_ and Ru/TiO_2_ the bathochromic shift with time is likely caused by the diffusion of electrons from the TiO_2_ bulk toward the surface. During this directional motion, electrons may recombine (non)radiatively with relatively immobile hole polarons, causing the PL spectrum to red-shift with time following excitation. The blue-shifted PL spectra and smaller bathochromic shift with time for Ru/TiO_2_ relative to TiO_2_ indicate surface PL quenching, likely due to charge transfer from the TiO_2_ surface into the Ru nanoclusters. When deposited on SiO_2_ and excited at 532 nm, Ru shows a strong emission. The PL of Ru when deposited on TiO_2_ is completely quenched, demonstrating interfacial charge separation following photoexcitation of the Ru nanoclusters with a close to unity quantum yield. The nature of the charge-transfer phenomena is discussed, and the obtained insights indicate that Ru nanoclusters should be deposited on semiconducting supports to enable highly effective photo(thermal)catalysis.

## Introduction

Due to an increasing energy demand and increasing amounts of greenhouse gases, interest in alternative fuel sources has increased dramatically in the past decades.^1,2^ Specifically, photocatalysis has gained interest as a promising “green” method to produce renewable fuels. Typically, in photocatalysis, a semiconductor is used to harvest solar energy to drive chemical reactions.^1−5^

Several recent studies have shown the promise of photoexciting metal nanoparticles to drive photocatalytic conversion at ambient conditions.^6−10^ A relatively new field combining the strengths of heterogeneous catalysis and photocatalysis is photothermal catalysis.^11−15^ Typically, metal nanoparticles are loaded on a metal oxide support, mostly in some form of TiO_2_. Importantly, the addition of photon energy to thermal energy enables us to (i) achieve significantly higher activities at relatively low temperatures and (ii) improve product selectivity by opening up new chemical reaction pathways, otherwise inaccessible.^16^

One explanation for the effect of light is that conversion is preceded by reactant adsorption (similar to “classical” heterogeneous catalysis), followed by light-induced electron transfer into the lowest unoccupied molecular orbital (LUMO) of surface adsorbates (the reactant), which weakens chemical bonds and thus lowers the activation energy for chemical conversion.^17,18^ Aside from these effects with the adsorbate, a variety of photoinduced processes can also occur between a metal nanoparticle and a metal oxide semiconductor onto which the particles are adsorbed,^19^ with the excitation wavelength likely playing an important role. Visible excitation of Au nanoparticles has been reported to lead to ultrafast hot electron transfer into TiO_2_.^20^ In the case of spectral overlap, Förster-type resonance energy transfer between the semiconductor and metal nanoparticle^8^ or between metal nanoparticles^21^ is also possible. Furthermore, it is essential to distinguish between few nanometer or smaller metal nanoclusters for which molecular-type electronic levels are well known^22−24^ and larger nanoparticles with a size-dependent plasmon resonance energy.^25^

Ultrafast spectroscopy is powerful to elucidate fundamental insights into light-induced mechanisms and dynamics. In our group, we have used time-resolved photoluminescence (PL) spectroscopy to understand the photodynamical processes in thin nanocrystalline anatase TiO_2_ films in aqueous media at different NaCl concentrations and at different pH values, enabling us to discriminate between bulk and surface charge carrier processes. The PL of the latter is red-shifted and sensitive to the environment. We also observed a red shift in the PL spectrum with time following photoexcitation, indicating directional charge diffusion from the TiO_2_ nanoparticle bulk toward its surface.^26^ Furthermore, significant insight has been gathered for commonly used silver or Ag nanoparticles. Especially, the intense PL of Au nanoclusters and small nanoparticles has been studied intensively,^23,27^ with the PL quantum yield increasing with a decreasing diameter,^28^ while Ag nanoclusters are also well known for their PL.^29^ However, for photothermal catalysis, one of the most effective nanoparticles consists of Ru.^30−32^ Very few photophysical studies for this system exist, and mechanistic insight regarding potential light-induced interfacial charge-transfer phenomena with a semiconductor support and the role of UV versus visible photoexcitation is limited.

In this work, we uncover the photoinduced charge carrier mechanisms of small Ru nanoclusters when decorating SiO_2_ or TiO_2_, two commonly applied supports in heterogeneous catalysis.^33−35^ We investigate through time-resolved PL spectroscopy the charge carrier processes occurring under ultraviolet (267 nm) or visible (532 nm) excitation and compare the phenomena to undecorated supports. The PL spectra of TiO_2_ and Ru/TiO_2_ are time-dependent and differ significantly. We extensively discuss the possible origin of the observed phenomena, with references to existing literature, and conclude that the photophysical mechanisms could contribute to the explanation of the photothermal catalytic phenomena. Finally, we also propose directions for further research.

## Methods

### Chemicals and Materials

The chemicals used in the described experiments include titanium(IV) oxide (TiO_2_, anatase nanopowder, <25 nm particle size, 99.7% trace metal basis), silicon dioxide (SiO_2_, nanopowder, 10–20 nm particle size, 99.5% trace metal basis), ruthenium(III) chloride hydrate (RuCl_3_·*x*H_2_O, 99.98% trace metal basis), poly(vinylpyrrolidone) (PVP, average molecular weight ∼55,000), methanol, demineralized water (Merck Milli-Q system, resistivity 18.2 MΩ·cm at 25 °C), sodium borohydride (NaBH_4_, purum p.a., ≥96%), acetone (for spectroscopy Uvasol), hydrogen peroxide solution (H_2_O_2_, 30% (w/w), puriss. p.a., reag. ISO, reag. Ph. Eur.), ammonium hydroxide solution (ACS reagent, 28.0–30.0% NH_3_ basis), barium sulfate (BaSO_4_, 1–4 μm Powder, 97%), UV-fused silica quartz substrates (Suprasil 2000, 12 mm × 30 mm × 1 mm, UQG optics Ltd., U.K.), and a fluorescence quartz cuvette (101-QS, Hellma Analytics, 10 mm × 10 mm optical path length). All chemicals were purchased from Sigma-Aldrich, except BaSO_4_ and ethanol, which were purchased from Alfa Aesar and Boom, respectively.

### Sample Preparation

Ru/TiO_2_ and Ru/SiO_2_ were prepared as follows: 0.103 g of RuCl_3_·*x*H_2_O and 0.523 g of PVP were dissolved in 200 mL of methanol and 160 mL of water. Then, 1 g of either TiO_2_ or SiO_2_ was added to the solution. After vigorous stirring for 1 h, 0.185 g of NaBH_4_ was added to the solution, yielding a color change into black. After stirring was continued for 2 h at room temperature, the temperature of the solution was elevated to 50 °C and stirring was continued further for 2 h. Then, the precipitate was intensively washed multiple times with Milli-Q water. Finally, the as-obtained product was dried overnight at 90 °C in air.

Unloaded and Ru-loaded TiO_2_ and SiO_2_ were coated on quartz substrates through a drop-casting procedure. First, the quartz substrates were cleaned through ultrasonication in a bath of acetone for 15 min, followed by ultrasonication in a bath of water for 15 min. Then, the substrates were rinsed with H_2_O and blow-dried with N_2_. To increase adhesion,^36^ the substrates were then treated for 30 min in a mixture of H_2_O, H_2_O_2_ (30 wt %), and NH_4_OH (28.0–30.0% NH_3_ basis) in a 5:1:1 ratio. Afterward, the quartz substrates were once more rinsed with water and put on a heating plate at 100 °C. Before drop-casting, the powders were brought in an aqueous suspension with a concentration of 10 g/L. After sonication for 30 min, the suspensions were drop-cast on the quartz substrates. After drying, the samples were treated in an oven in air at 200 °C overnight. As-prepared samples were stored in argon afterward.

### Characterization

Characterization of the samples took place using several techniques prior to the drop-casting step. To determine the dispersion and morphology of Ru on TiO_2_ and SiO_2_, high-angular annular dark-field (HAADF) images were collected through scanning transmission electron microscopy (STEM) measurements, which were performed using an FEI cubed Cs corrected Titan. For elucidation of the oxidation state of Ru, X-ray photoelectron spectroscopy (XPS) measurements were performed using a PHI Quantes scanning XPS/HAXPES microprobe with a monochromatic Al Kα X-ray source (1486.6 eV). Diffuse reflectance spectroscopy was performed using the deuterium lamp of an Avantes AvaLight-DH-S-BAL light source. An Avantes AvaSpec-2048 spectrometer was used to determine the diffuse reflectance spectra of the different samples. BaSO_4_ was used as a reference sample. The Kubelka–Munk plots *F*(*R*) were calculated from these diffuse reflectance spectra through the following formula^37^
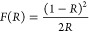
1where *R* is the measured reflectance. These Kubelka–Munk plots correlate with the absorbance spectra of the samples. Finally, to elucidate the difference in crystallinity between the TiO_2_ used in this study compared to TiO_2_ used in our previous study,^26^ we performed X-ray diffraction (Bruker D2 Powder) using the Cu Kα line under an accelerating voltage of 30 kV.

### Time-Resolved PL Experiments

The experimental setup used for photoluminescence experiments has been described in detail in previous work.^26^ Briefly, the output of a Fianium laser (FP-532-1-s, 532 nm center wavelength, 300 fs pulse duration, and 80.37 MHz repetition rate) was used as a light source. For experiments performed with λ_exc._ = 532 nm, the output was attenuated to 25 mW. For experiments with λ_exc._ = 267 nm, a second harmonic UV signal was generated by focusing 700 mW into a 3 mm thick β-BaB_2_O_4_ crystal (Newlight Photonics) using a 20 cm focal length quartz length and recollimated after the second harmonic generation using a 20 cm focal quartz length. The output was sent by three dichroic mirrors (Thorlabs, MBI-K04) through an FGUV11-UV filter (Thorlabs) to remove the residual 532 nm component to the sample. The λ_exc._ = 267 nm and λ_exc._ = 532 nm experiments were performed using a power of 27 and 8.2 μW, respectively. The sample was kept in a sealed fluorescence quartz cuvette (101-QS, Hellma Analytics, 10 mm × 10 mm optical path length), cleaned with ethanol, and filled with argon.

The PL signals emitted from the layers on quartz were collected and focused on the input of a spectrograph (Acton SP2300, Princeton Instruments, 100 μm slit width, 50 lines/mm grating blazed at 600 nm) with two 2 in. focal glass lenses (50 mm focal length). The PL signal of the UV-fused silica quartz substrates was verified to be negligible for both 267 and 532 nm excitations. In the case of photoexcitation at 532 nm, the PL signal was sent through a 570 nm long-pass filter to avoid the 532 nm light inevitably scattered by the sample to enter the streak camera setup. The slit in front of the photocathode of the streak camera was set at 180 μm, yielding a time resolution of 30 ± 1 ps at a time range of 5 (*i.e.*, a time window of 2 ns) and 15 ± 1 ps at a time range of 3 (*i*.*e*., a time window of 200 ps). Prior to the time-resolved PL experiments, the spectral calibration was checked and adapted if necessary using a Hg/Ar calibration lamp (Oriel, LSP035). Furthermore, the PL spectra were corrected for the spectral sensitivity of the setup measured using a calibrated blackbody lamp (Ocean Optics, HL-2000). The time windows used were either 2 ns (*i*.*e*., time range of 5) or 600 ps (*i*.*e*., time range of 3). The PL decay was verified to remain constant during the integration time (Figure S1), although the amplitude decreased over the course of hours.

The open-source program Glotaran^38^ was used to perform global analysis, analogous to our earlier work on the nature of PL in nanostructured TiO_2_^26^ and commonly used to account for the spectral overlap of coexisting species and to disentangle their individual spectra and dynamics.^39^ The spectrotemporal PL behavior could be described with two pathways, with the exception for TiO_2_, where a description of three pathways is more accurate (see the Results and Discussion section). Initial fitting and determination of τ_1_ and τ_2_ (and possibly τ_3_) values were realized with the data of a time range of 5. By fixing the value(s) of τ_2_ (and τ_3_), the value of τ_1_ was determined more accurately using data in the time range of 3. To determine the final lifetime values, multiple iterations were performed until the point that the values stabilized (*i*.*e*., changed less than the error).

## Results and Discussion

### Material Characterization

XRD analysis confirms that the applied TiO_2_ consists of a major portion of anatase and a minor portion of rutile (see Figure S2). Note that Degussa P25, a combination of roughly 80% anatase and 15% rutile (the remaining 5% can be attributed to an amorphous phase),^40^ shows a higher photocatalytic activity than either pure rutile or pure anatase TiO_2_.^41^ The PL spectra of anatase and rutile TiO_2_ are known to differ,^42−44^ while the interface of rutile and anatase was reported to promote light-induced charge separation and the photocatalytic activity.^45^

**Figure 1:**
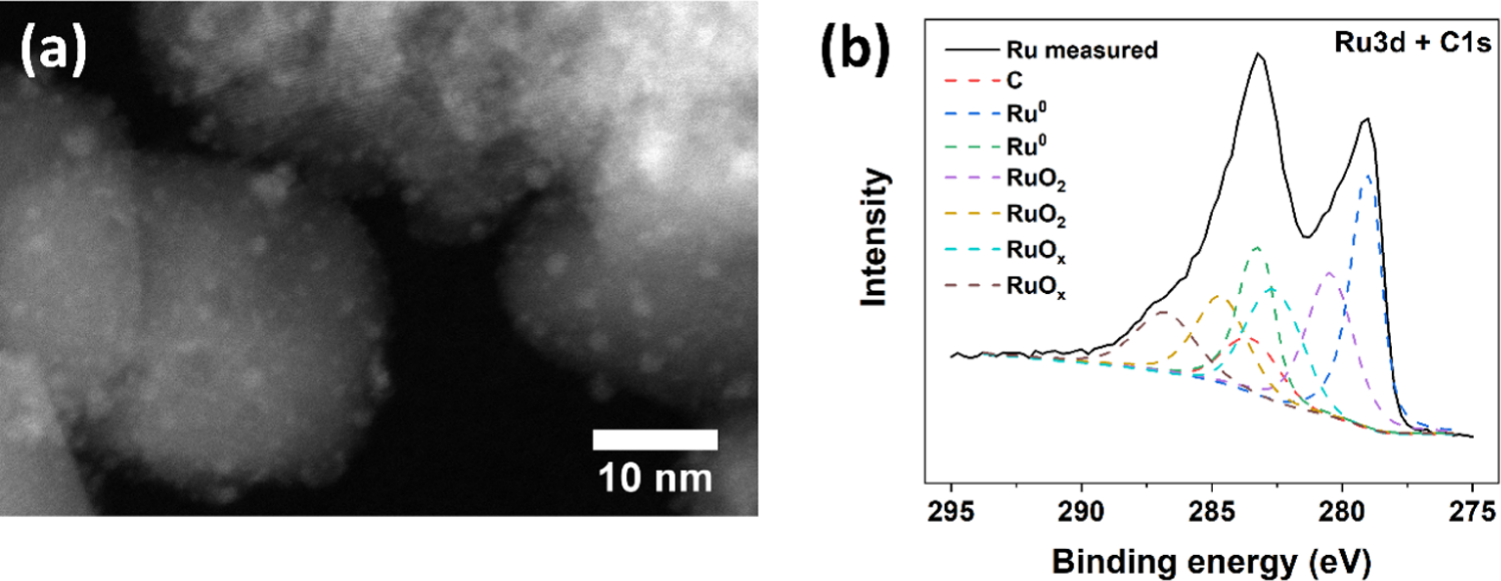
(a) HAADF-STEM image of Ru deposited on TiO_2_ and (b) X-ray photoelectron spectrum of the Ru particles including the spectral deconvolution.

Figure 1a presents the annular dark-field image obtained through scanning transmission electron microscopy of Ru/TiO_2_, showing a nanocrystalline structure consisting of TiO_2_ nanoparticles with a size of ∼20 nm. The TiO_2_ surface is mostly decorated with 1–2 nm diameter Ru nanoparticles, with outliers at 0.5 and 3 nm; such small nanoparticles are often referred to as nanoclusters.^27^ On SiO_2_, Ru nanoparticles are present with a size distribution ranging from 0.5 to 5 nm (Figure S3). They are also less dispersed and form aggregates. XPS shows that the Ru nanoclusters consist of metallic Ru (*ca*. 40%) but are also partly oxidized (Figure 1b and Table S1). Although it is hard to exactly elucidate the distribution between oxidized and reduced Ru, it is likely that exposure to air results in a partial oxidation of the surface. Thus, we postulate that the Ru nanoparticles are possibly deposited onto the TiO_2_ or SiO_2_ as tiny core–shell particles, with a metallic core and a thin oxidized shell.

The Kubelka–Munk plots of TiO_2_, SiO_2_, Ru/TiO_2_, and Ru/SiO_2_ are shown in Figure S4. Analogous to other studies,^46^ TiO_2_ is only able to absorb light <400 nm. This agrees with literature values for a band gap of 3.0 eV for rutile and 3.2 eV for anatase.^46,47^ Since SiO_2_ is an insulator, a negligible signal is observed in the Kubelka–Munk plot. The Ru nanoparticles allow for visible light absorption of Ru/TiO_2_ and Ru/SiO_2_. This absorption can originate from both metallic Ru and from RuO_2_. Very small (few nanometer diameter) metal nanoclusters are known to show molecular-type electronic transitions.^27^ RuO_2_ has a band gap of 2.3 eV,^48^ which may be larger here due to the small diameters of the nanoparticles, likely giving rise to quantum confinement effects. Based on Figure S4, TiO_2_ and Ru/TiO_2_ are expected to absorb laser light with an excitation wavelength of 267 nm strongly and Ru/SiO_2_ mildly. Only the Ru nanoclusters and particles should be able to absorb the 532 nm laser light.

### Time-Resolved Photoluminescence (PL) Studies

**Figure 2:**
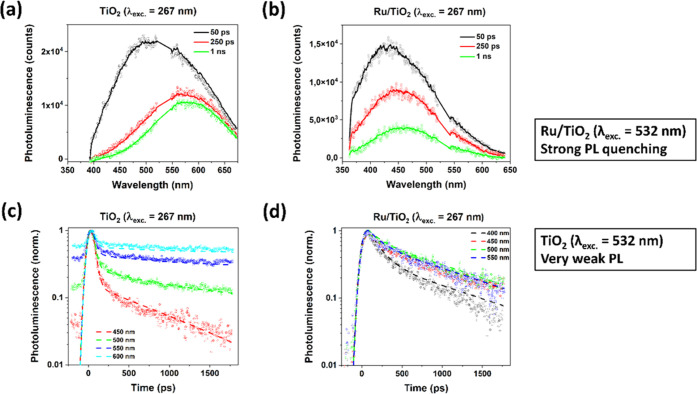
PL spectra at 50 ps, 250 ps, and 1 ns after 267 nm of a 300 fs excitation of nanostructured TiO_2_ in Ar (a, with the 2 ns time window of the synchroscan unit; a nondecaying PL component in the near-IR could not be resolved) and Ru/TiO_2_ in Ar (b); panels (c) and (d) show normalized PL decays at selected wavelengths. The solid lines are fits from global analysis using a parallel model with 2 or 3 components (see Table 1). Data around 532 nm have been removed because of the scattering of residual laser light, while potential PL < 350 nm was blocked by the two 2 in. glass lenses used for collecting the PL.

To explore a potential role of UV versus visible photoexcitation in the charge carrier dynamics, as well as the occurrence of interfacial charge separation between the TiO_2_ and the Ru nanoclusters, time-resolved photoluminescence (PL) studies were performed by excitation with 300 fs pulses with a center wavelength of either 267 or 532 nm. Figure 2a presents the PL spectra at 50, 250, and 1 ns after a 267 nm excitation of a nanoporous TiO_2_ film in an Ar atmosphere, showing a decay at a picosecond–nanosecond time scale coinciding with a bathochromic shift with time from *ca*. 505 to 580 nm. The PL spectrum at 50 ps is substantially blue-shifted compared to that at 250 ps, indicating a different physical origin of the first. The red shift continues from 250 ps to 1 ns although less substantial. The red-shifted PL spectra and the stronger bathochromic shift with time in the present work relative to our earlier study on nanoporous anatase TiO_2_ in various aqueous solutions^26^ are likely due to differences in the crystalline phase (see Figure S2 for XRD), preparation method, and/or environment. In our previous work, we assigned this bathochromic shift with time to electron diffusion from the TiO_2_ bulk toward the surface. This process likely occurs through multiple trapping and detrapping of electrons that are relatively mobile and likely move via a hopping-type process.^49^ During this process, they may recombine (non)radiatively with relatively immobile hole polarons. This directional electron diffusion can also explain the wavelength dependency in the PL decay observed (Figure 2c). The PL at the highest photon energies, presumably primarily originating from bulk recombination, likely decays the fastest due to electron diffusion competing with the PL, hence lowering the PL lifetime. On the contrary, electron diffusion close to the possibly deeper trap states close to or at the TiO_2_ surface^26^ is likely slower and therefore less competitive to radiative decay. This also explains why the red shift in the PL spectrum especially occurs at early times, as evident from, *e*.*g*., the spectra at 50 and 250 ps in Figure 2a. At 250 ps, a major fraction of the electrons have reached the TiO_2_ surface, explaining the minor red shift from 250 ps to 1 ns and the appearance of a nondecaying component. The latter causes the background signal (before *t* = 0 ps) to increase due to the back sweep of the streak camera used for PL detection. With the time window of the synchroscan unit (2 ns), this leads to a nondecaying PL component in the near-IR that could not be resolved. Due to the very low intensity of the PL signal, measurements at a lower photoexcitation repetition rate with single photon counting detection are unfeasible and have therefore not been performed. If such experiments would be feasible, the absence of the streak camera back streak in single photon counting detection can be expected to slightly affect the slow decay above *ca*. 550 nm. In the case of >1–2 ns PL lifetimes, the back streak yields a slightly slower decay than reality.^50^ However, the streak camera is perfectly suitable to catch the subnanosecond decay at higher photon energies (see Table 1 for lifetimes), and this will therefore not be affected. Even for the slowest PL decay observed for TiO_2_ following a 267 nm excitation, the extrapolated PL at 12 ns relative to the maximum PL intensity observed around 500 nm is very weak, 5% of that value at 600 nm (Figure S5). At the other PL wavelengths, this percentage is lower. Charge accumulation due to long-lived carriers in deep trap states, which is hard to completely eliminate in a metal oxide semiconductor, is hence minor.

**Table 1:** PL Lifetimes from Global Analysis Using a Parallel Decay Model with 2 or 3 Components

	τ_1_ (ps)	τ_2_ (ps)	τ_3_ (ps)
TiO_2_ in Ar, 267 nm exc.	25.5 ± 0.07	954.8 ± 2.8	∞
Ru/TiO_2_ in Ar, 267 nm exc.	733.9 ± 12.3	1113 ± 1.2	
Ru/SiO_2_ in Ar, 532 nm exc.	200.1 ± 1.2	985.3 ± 1.9	
Ru/TiO_2_ in Ar, 532 nm exc.	strong PL quenching		

Figure 2b shows the PL spectra for Ru/TiO_2_ in Ar after a 267 nm excitation. Compared to TiO_2_ (Figure 2a; see also Figure S7), the PL spectra are clearly blue-shifted. Considering the minor differences between the PL of Ru/SiO_2_ and SiO_2_ at a 267 nm excitation (Figure S6), a major contribution of the Ru nanoclusters to the PL is unlikely under these conditions. As SiO_2_ is a wide-band-gap semiconductor and does not absorb at 267 nm (see also Kubelka–Munk plots in Figure S4), the PL from the SiO_2_ is likely a result of the sub-band-gap excitation followed by emission from trap states. The blue emission observed agrees with the earlier work, in which the PL was assigned to defects.^51^ The minor red shift and broadening of the PL spectrum observed for Ru/SiO_2_ compared to that for SiO_2_ is likely a result of the impact of Ru nanoparticles on trap states in the SiO_2_, giving rise to the PL signal. The lack of substantial PL from the Ru nanoparticles upon UV excitation also agrees with the absence of a more intense PL signal for Ru/TiO_2_ compared to that of TiO_2_. The PL spectra in Figure 2b are hence likely predominantly a result of TiO_2_ photoexcitation. Interestingly, the PL spectra of Ru/TiO_2_ are blue-shifted relative to those of TiO_2_, and the bathochromic shift with time from *ca*. 435 to 460 nm is also largely reduced compared to bare TiO_2_ (Figure 2a), indicating that Ru nanoclusters quench in particular the surface PL of TiO_2_. The absence of a significant difference in the PL intensity of Ru/TiO_2_ relative to TiO_2_ at shorter wavelengths excludes ultrafast (*i*.*e*., within the instrumental response time) interfacial photoinduced charge separation, although this may occur to some degree at a nanosecond time scale after photoexcitation for charge carriers that have succeeded to diffuse to the Ru/TiO_2_ interface. Figure 2d shows the decay at the selected PL wavelengths. Again, a gradual increase in PL lifetime is observed with lowering the photon energy. Two important differences are noticeable relative to that of bare TiO_2_. First, the fast component especially pronounced at higher photon energies is absent, which is likely a result of the surface functionalization as discussed below. Also, the nondecaying component observed for TiO_2_ at low photon energies is absent, likely as a result of Ru nanoclusters quenching the surface PL of TiO_2_.

Upon switching the excitation wavelength from 267 to 532 nm, the PL behavior changes drastically. As can be expected, the illumination of TiO_2_ only results in scattering of the 532 nm pulses and no detectable PL (see Figure S8). The strong PL signal centered around 590 nm observed for Ru/SiO_2_ in Ar (Figure S9a) decaying in a picosecond to nanosecond time window hence primarily originates from photoexcitation of the Ru nanoclusters, which are also responsible for the absorption of visible light (Figure S4). Note that the illumination of SiO_2_ at 532 nm does not give any detectable PL (Figure S8). Figure S9b shows a weak wavelength dependency of the PL decay of Ru/SiO_2_, possibly due to some structural inhomogeneity. This PL behavior is in agreement with the literature on a few nanometer size metal nanoclusters, for which molecular-type electronic levels are well known.^22−24^ For 2 nm diameter Ru nanoclusters, a broad PL band around 560 nm was reported,^52^ while *ca*. 1.5 nm diameter Ru nanoclusters were observed to show a broad PL band around 460 nm.^53^ In contrast, despite the absorption of the Ru nanoclusters at 532 nm (Figure S4) and the PL observed in the present work on insulating SiO_2_ (Figure S6) and in earlier work for 1.5–2 nm diameter Ru nanoclusters in solution,^52,53^ no PL could be detected for Ru/TiO_2_. This striking difference indicates the PL quenching of the Ru nanocluster excited states, most likely by ultrafast interfacial charge separation with the TiO_2_. Förster-type resonance energy transfer^8^ from the Ru nanoclusters toward the TiO_2_ is unlikely because of the lack of spectral overlap. The occurrence of charge separation agrees with density functional theory studies, reporting photoinduced electron transfer from excited Ru nanoclusters into TiO_2_.^54^ The present work shows that this light-induced interfacial charge separation process likely occurs within the instrumental response time of the streak camera, either during photoexcitation^55^ of the Ru nanoclusters or shortly thereafter on a femtosecond to early picosecond time scale.

**Figure 3:**
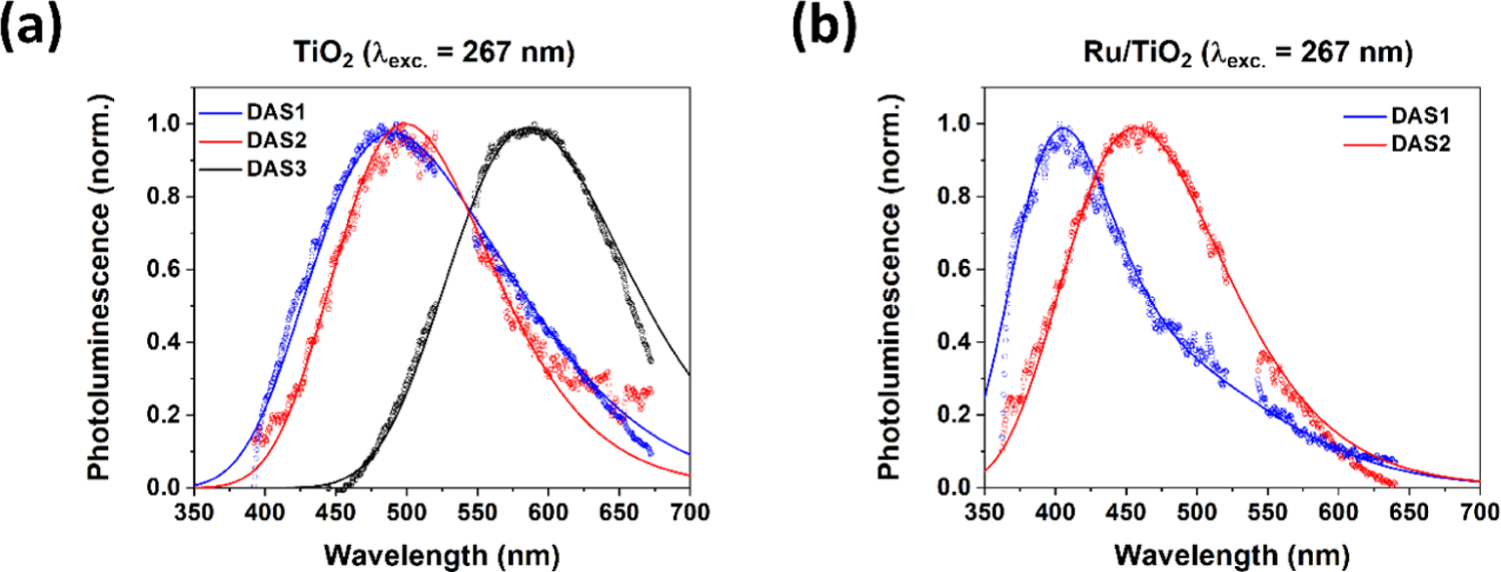
Normalized DAS of TiO_2_ in Ar (a) and Ru/TiO_2_ in Ar (b) under a 267 nm excitation. The solid lines present the sum of Gaussian functions with parameters presented in Table 2.

Global analysis demonstrates that the spectrotemporal PL behavior is well described by a parallel decay model, analogous to our earlier work on nanostructured anatase TiO_2_ in different aqueous solutions.^26^ A parallel model instead of a sequential model has been chosen because of the full development of the PL signal within the instrumental response time and the absence of a subsequent increase in signal. Note that although this model is likely a simplification of the reality, it describes all PL data well as apparent from the fits included as lines in Figures 2, S5, and S8. Figure 3 presents the normalized decay-associated spectra (DAS) obtained from global analysis using a parallel decay model; that is, DAS1 decays with τ_1_, DAS2 decays with τ_2_, and (only for TiO_2_ at a 267 nm excitation) DAS3 decays with τ_3_. Table 1 presents the minimum number of parallel decay processes needed for a good fit and obtained lifetimes. The spectrotemporal behavior of TiO_2_ at a 267 nm excitation is well described by a parallel model with three components, while for Ru/TiO_2_ at these conditions, we only need two components, likely due to the TiO_2_ surface PL quenching by the Ru nanoclusters. A good fit for the PL of Ru/SiO_2_ at a 532 nm excitation is obtained by using a parallel decay model with two components (Figure S10). The obtained lifetimes (Table 1) are comparable to values in the literature for a few nanometer size Au nanoclusters.^56,57^

An important question to answer is whether the DAS indeed consists of one component, *i*.*e*., it presents a single photophysical process, or whether a second (minor) component is present. This would be applicable in the case where the DAS corresponds to more than one photophysical decay process. Spectral deconvolution shows that the DAS are well described by Gaussian functions, with corresponding parameters presented in Table 2. For TiO_2_ at a 267 nm excitation, both DAS2 and DAS3 are well described by single Gaussians, centered at 2.48 and 2.11 eV, respectively. DAS1 is predominantly described by a PL band centered at 2.55 eV and a shoulder (14%) of the 2.11 eV band. Similarly, for Ru/TiO_2_ at a 267 nm excitation, DAS1 can be deconvoluted into two Gaussians centered at 3.07 eV and a shoulder (21%) at 2.45 eV, while for DAS2, a single Gaussian centered at 2.71 eV is sufficient. For Ru/SiO_2_ at a 532 nm excitation, DAS1 is well described by a single Gaussian centered at 2.11 eV, while DAS2 has in addition to this band a tail (25%) centered at 1.79 eV, which likely arises from some structural inhomogeneity.

**Table 2:** Parameters of the Gaussian Functions Used for the Spectral Deconvolution of the DAS

	DAS1 (τ_1_)	DAS2 (τ_2_)	DAS3 (τ_3_)
TiO_2_ in Ar, 267 nm exc.	2.55 eV (σ = 0.32 eV, 86%)	2.48 eV (σ = 0.27 eV)	2.11 eV (σ = 0.22 eV)
	2.11 eV (σ = 0.22 eV, 14%)		
Ru/TiO_2_ in Ar, 267 nm exc.	3.07 eV (σ = 0.28 eV, 79%)	2.71 eV	
	2.45 eV (σ = 0.29 eV, 21%)		
Ru/SiO_2_ in Ar, 532 nm exc.	2.11 eV (σ = 0.16 eV)	2.11 eV (σ = 0.16 eV, 75%)	
		1.79 eV (σ = 0.16 eV, 25%)	
Ru/TiO_2_ in Ar, 532 nm exc.	strong PL quenching		

### Discussion and Proposed Photophysical Models

**Figure 4:**
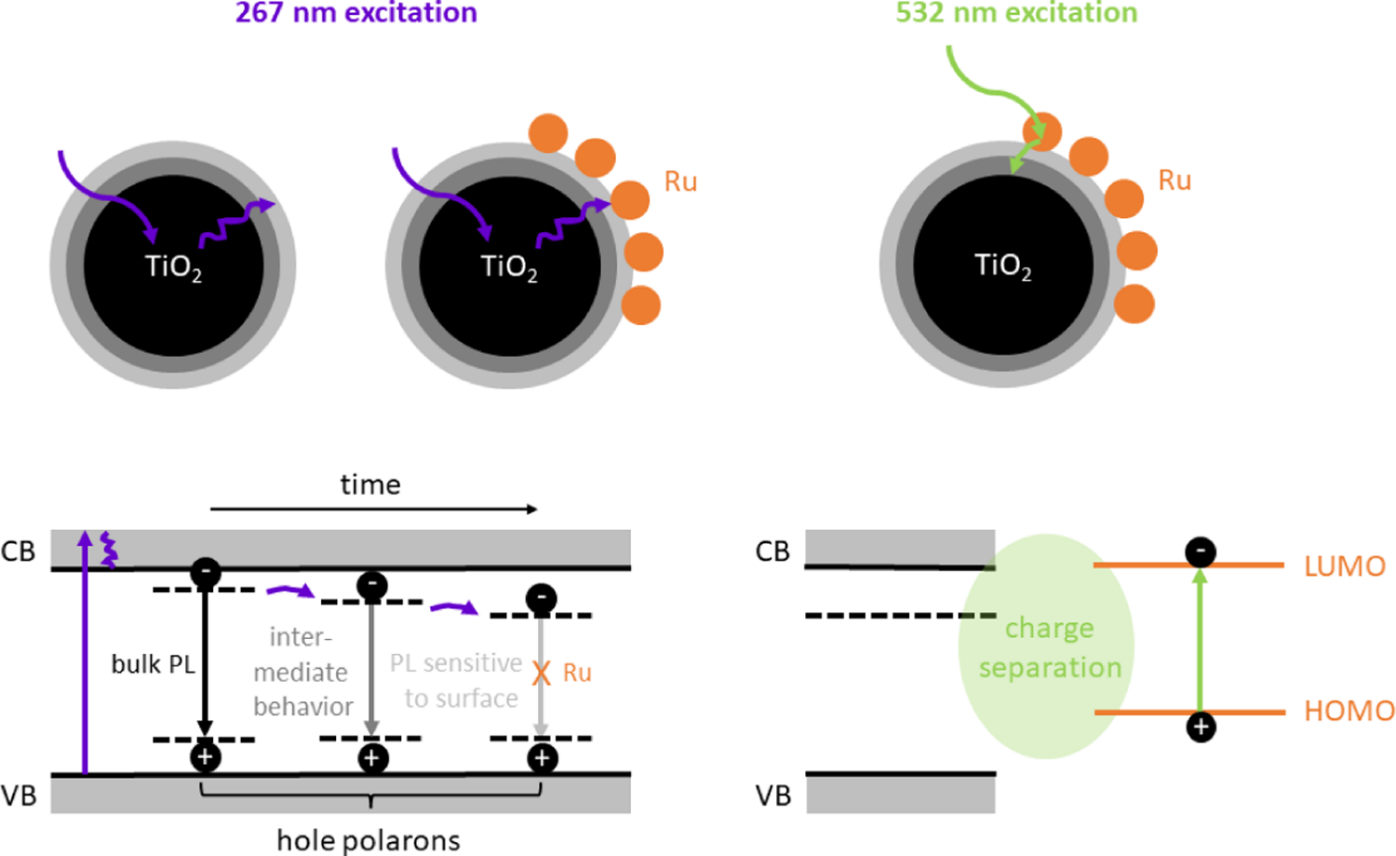
Proposed photophysical models for 267 and 532 nm excitations. The first primarily leads to the excitation of electrons from the TiO_2_ valence band (VB) into the conduction band (CB), followed by diffusion toward the surface and recombination with relatively immobile hole polarons in time. Ru nanoclusters quench the TiO_2_ surface PL. At 532 nm, mainly the Ru nanoclusters are excited, which decay by interfacial charge separation with the TiO_2_.

In Figure 4, we propose photophysical models for the processes following photoexcitation of TiO_2_ and Ru/TiO_2_, highlighting the differences between 267 and 532 nm excitations. The first mainly leads to photoexcitation of the TiO_2_, whereas photoexcitation of the Ru nanoclusters is minor or negligible under these conditions. Since the photon energy (4.64 eV) exceeds the TiO_2_ band gap, photoexcitation initially leads to the generation of hot or nonthermalized electrons, which thermalize by electron–phonon coupling reported to occur in <50 fs.^58^ The interaction of electrons with immobile hole polarons may lead to self-trapped excitons, although these have not been included in Figure 4 because of the <5% quantum yield of this process at room temperature.^59^ During the 300 fs photoexcitation pulse, electrons and holes trapped in shallow bulk and surface traps are likely generated,^60,61^ which can explain why for the spectral deconvolution of DAS1 of both TiO_2_ and Ru/TiO_2_ two Gaussians are needed (Table 2), indicating two physical origins of DAS1. The dominant Gaussian at the highest PL photon energy likely presents bulk charge recombination, and the second weaker Gaussian at the lowest PL photon energy presents surface charge recombination. As the mobility of electrons in TiO_2_ is likely at least 10 times higher than that of holes,^62^ photoexcitation can be expected to be mainly followed by the diffusion of electrons. Based on an electron diffusion coefficient of 2 × 10^–5^ cm^2^/s for nanostructured TiO_2_,^63^ a 1–2 ns diffusion time from the bulk toward the surface can be estimated. During this multiple (de)trapping process,^49^ electrons likely gradually relax into deeper traps,^26^ causing a bathochromic PL shift with time. The observation that the bathochromic shift in Figure 2a mainly occurs in the first 250 ps after excitation and slightly further from 250 ps to 1 ns indicates that this diffusion predominantly occurs within 250 ps and slightly beyond this time window. Based on this assignment and our earlier work,^26^ we cautiously assign DAS2 to a decay process with intermediate behavior between bulk electron–hole recombination and recombination sensitive to surface termination (DAS3). The blue-shifted PL spectra for Ru/TiO_2_ relative to TiO_2_, the diminished bathochromic shift with time, and the lack of a necessity to include DAS3 in the global analysis clearly demonstrate that the presence of Ru nanoclusters quenches especially the surface PL of the TiO_2_.

The quenching of the low-energy PL of TiO_2_ induced by the Ru nanoclusters can be assigned to several effects. First, the Ru nanoclusters may introduce new trap states within the TiO_2_ band gap at or near the surface.^34,54,64^ Second, the Ru nanoclusters may passivate existing TiO_2_ surface trap states. For a (101) TiO_2_ surface, deep electron and hole traps have been assigned to undercoordinated Ti_5c_^3+^ and O_2c_^–^ sites, on which the Ru nanoclusters can be expected to have a major impact. A third possibility is that an ultrathin RuO_2_ shell around the Ru nanocluster (see the XPS analysis in Figure 1 and Table S1) accepts photoinduced holes from the TiO_2_, as reported earlier.^65−67^ As holes in nanocrystalline TiO_2_ are relatively immobile compared to electrons,^62^ this process is likely most relevant for holes trapped at or close to the TiO_2_ surface. The resulting low quantity of surface hole polarons will have consequences for electrons that succeed to diffuse from the bulk toward the TiO_2_ surface, as they could not recombine (non)radiatively with trapped holes any longer. Considering the ultrathin RuO_2_ shell around the Ru nanocluster (Figure 1), we expect that the latter scenario could play a significant role here, which can also explain the difference in τ_1_ values between Ru/TiO_2_ (733.9 ± 12.3 ps) and TiO_2_ (25.5 ± 0.07 ps). A lower quantity of surface hole polarons for Ru/TiO_2_ implies less surface electron–hole recombination competing with (non)radiative recombination in the bulk of the TiO_2_ nanoparticle and therefore a longer τ_1_ value. The longer τ_1_ and τ_2_ values observed here for Ru/TiO_2_ compared to those for TiO_2_ indicate that electron transfer from photoexcited TiO_2_ into the Ru is less likely, as such an electron-transfer process can be expected to decrease τ_1_ and τ_2_.

At a 532 nm photoexcitation, the situation is entirely different. In this case, the Ru nanoclusters are mainly responsible for the emission observed on the insulating SiO_2_ support (Figure S9), and the PL is well described by a parallel model with two lifetimes (200.1 ± 1.2 and 985.3 ± 1.9 ps). This PL behavior likely originates from molecular-type LUMO and highest occupied molecular orbital (HOMO) electronic levels well known for a few nanometer size metal nanoclusters.^22,23^ The PL spectrum agrees with earlier work on Ru nanoclusters, reporting a broad PL band around 560 nm for 2 nm diameter Ru nanoclusters.^52^ The obtained PL lifetimes are also comparable to literature values for a few nanometer size Au nanoclusters.^56,57^ The biphasic decay may originate from a distribution in Ru nanocluster diameters, oxidation states, nanocluster aggregation, and/or distance-dependent Förster resonance energy transfer between the Ru nanoparticles.^68^ In contrast to present results on Ru/SiO_2_ and earlier work on unsupported 1.5–2 nm Ru nanoclusters in solution,^52,53^ the PL of Ru/TiO_2_ is strongly quenched, indicating ultrafast interfacial charge separation following photoexcitation of the Ru nanoclusters. Based on the striking difference in PL quenching between the Ru nanoclusters on insulating SiO_2_ and TiO_2_, we assume that the role of the thin RuO_2_ shell likely present at the surface of the Ru nanoclusters (Table S1) is not the major factor in PL quenching. The RuO_2_ shell is likely thin enough to allow charge tunneling^69,70^ between the Ru core of the nanocluster and the TiO_2_ substrate. Based on the UV–vis and PL spectra, the HOMO–LUMO energy gap of the Ru nanoclusters is estimated to equal ∼2.4 eV and likely depends on the diameter. Density functional theory studies on Ru_10_ nanoclusters on anatase TiO_2_ (101) immersed into water indicate that photoexcitation of the Ru nanocluster is followed by electron transfer into the TiO_2_.^54^ Photoexcitation of 1–3 nm size Au nanoclusters^71^ and 10 nm diameter Au nanoparticles^72,73^ was also reported to result in electron transfer into TiO_2_. Based on these studies, we cautiously propose that photoinduced interfacial charge separation may occur by electron transfer from the LUMO of the Ru nanocluster, through the ultrathin RuO_2_ shell, into the TiO_2_ conduction band. The strong PL quenching indicates that the quantum yield for light-induced charge separation is likely close to unity. The nature of the charge-transfer process will depend on the Ru LUMO energy level, relative to the CB minimum of TiO_2_. In case the LUMO level is equal to or higher in energy, electron transfer from Ru into TiO_2_ is allowed. Alternatively, hole transfer following photoexcitation of the Ru from the HOMO into, *e*.*g*., a surface trap state of the TiO_2_ may occur. The distribution in Ru particle diameters and oxidation states may well result in a distribution in HOMO and LUMO energy levels and, as a result, alter the photoinduced interfacial charge-transfer mechanism.

The major impact of UV versus visible photoexcitation on the interface processes uncovered in the present work has important consequences for the nanostructural design of Ru/TiO_2_ photocatalysts and the choice of illumination source. The light sources used in photocatalytic and thermal studies are diverse and typically range from a solar simulator to a Hg or Xe lamp or light-emitting diode (LED).^74^ Importantly, the contribution of UV versus visible light varies for these sources, while the present work clearly demonstrates key differences. The TiO_2_ surface PL quenching observed for a 267 nm excitation, likely due to the transfer of surface hole polarons into the RuO_2_, can be considered as a cocatalytic effect in which the surface oxidation of the Ru nanocluster or particle likely plays an important role. As a key process under these conditions involves the generation of mobile electrons in the TiO_2_ nanoparticle bulk, which first need to diffuse toward the surface before utilization in a photocatalytic process is possible and during which process losses occur, this implies a relatively low quantum yield for light-induced charge separation. In contrast, illumination at 532 nm predominantly excites the Ru nanoclusters, which results in ultrafast charge separation with the TiO_2_ with a likely close to unity quantum yield.

Outcompeting intrinsic decay processes within metal nanoparticles by interfacial charge separation with a metal oxide semiconductor can be challenging,^18,75^ although light-induced interfacial charge separation could occur during photoexcitation via direct electron transfer.^76,77^ The efficient charge separation observed in the present work likely results from the relatively slow molecular-type excited-state decay dynamics of the Ru nanoclusters (Table 1), enabling light-induced interfacial charge transfer to outcompete intrinsic excited-state decay processes. To the best of our knowledge, this is the first time that time-resolved PL spectroscopy studies have been performed on Ru-loaded TiO_2_ to elucidate the charge carrier mechanisms induced by light absorption. Considering the key role of the photoexcitation wavelength in the mechanism and quantum yield of interfacial charge separation unraveled in the present work is essential in the design of efficient Ru/TiO_2_ photocatalysts.

## Conclusions

In this work, we have uncovered the light-induced processes for a few nanometer size Ru nanoclusters deposited onto nanocrystalline TiO_2_ by time-resolved PL spectroscopy, with a major role of the photoexcitation wavelength in the mechanism and quantum yield of light-induced charge separation. The Ru nanoclusters cause (i) quenching of surface PL of TiO_2_ following photoexcitation at 267 nm and (ii) show no PL when deposited on TiO_2_ and excited at 532 nm, which in both cases can be explained by charge-transfer phenomena occurring at the Ru/TiO_2_ interface. We anticipate the role of a thin RuO_2_ shell in the phenomena upon a 267 nm excitation, whereas the Ru metal core plays an important role at a 532 nm excitation.

Currently, we are expanding the time-resolved PL setup to investigate how *in situ* photothermal conditions, including a reductive gaseous atmosphere (affecting the Ru oxidation state and inducing the presence of molecular adsorbates), influence photoinduced interfacial charge separation, to develop a mechanistic understanding in the possible synergy of light and elevated temperature in photothermal catalysis.

## Supporting Information Available

Normalized PL decay of Ru/SiO_2_ in Ar recorded at the beginning of integration and after 3 h of illumination; XRD pattern of TiO_2_; HAADF-STEM image and XPS spectrum of Ru/SiO_2_; XPS analysis of Ru/TiO_2_ and Ru/SiO_2_; Kubelka–Munk plots of TiO_2_, SiO_2_, Ru/TiO_2_, and Ru/SiO_2_; PL spectra of SiO_2_ and Ru/SiO_2_ in Ar with λ_exc._ = 267 nm; comparison of PL spectra between TiO_2_ and Ru/TiO_2_ in Ar and SiO_2_ and Ru/SiO_2_ in Ar with λ_exc._ = 267 nm; number of photons detected for TiO_2_ and SiO_2_ in Ar with λ_exc._ = 532 nm; PL spectra of Ru/SiO_2_ in Ar with λ_exc._ = 532 nm; and normalized DAS spectra of Ru/SiO_2_ in Ar with λ_exc._ = 532 nm (PDF)
