# Supplementary material for: Photophysical Characterization of Ru Nanoclusters on Nanostructured TiO2 by Time-Resolved Photoluminescence Spectroscopy
Source: J Phys Chem C Nanomater Interfaces. 2023 Jul 15;127(29):14353–62. doi: 10.1021/acs.jpcc.3c04075 (PMC10388344; doi:10.1021/acs.jpcc.3c04075)
Supplement: Supplementary file 1 — jp3c04075_si_001.pdf [file jp3c04075_si_001.pdf]

Supporting information

Photophysical Characterization of Ru Nanoclusters on Nanostructured TiO_2 by Time-Resolved Photoluminescence Spectroscopy

*Kasper Wenderich[1] *, Kaijian Zhu[1], Yibin Bu[2], Frans D. Tichelaar[3], Guido Mul[1], Annemarie Huijser[1] **

**k.wenderich@utwente.nl & j.m.huijser@utwente.nl*

[1]Photocatalytic Synthesis Group, Faculty of Science and Technology, MESA+ Institute for Nanotechnology, University of Twente, P.O. Box 217, 7500 AE Enschede, the Netherlands

[2]Nanolab, MESA+ Institute for Nanotechnology, University of Twente, P.O. Box 217, 7500 AE Enschede, the Netherlands

[3]Kavli Institute of Technology, Quantum Nanoscience, Delft University of Technology, 2628 CJ Delft, the Netherlands

Figures S1 and S5-9 present supporting time-resolved photoluminescence (PL) data and decay associated spectra (DAS), which are detailed further in the main text. Figure S2a shows the XRD pattern of TiO_2, including the reference patterns of anatase and rutile.[1-2] The XRD pattern of SiO_2 (Figure S2b) shows an amorphous layer structure. Figure S3 shows the HAADF-STEM image and X-ray photoelectron spectrum of Ru/SiO_2. Table S1 shows the atomic percentages of the oxidation states of Ru as detected by XPS. Peak-fitting was performed on the Ru3d core spectra. The area percentages were used to determine the atomic percentages of the oxidation states of Ru. A correction took place for the overlap with the C1s core spectra, which overlaps with the Ru3d core spectra. Figure S4 shows the Kubelka-Munk plots of TiO_2, SiO_2, Ru/TiO_2 and Ru/SiO_2.

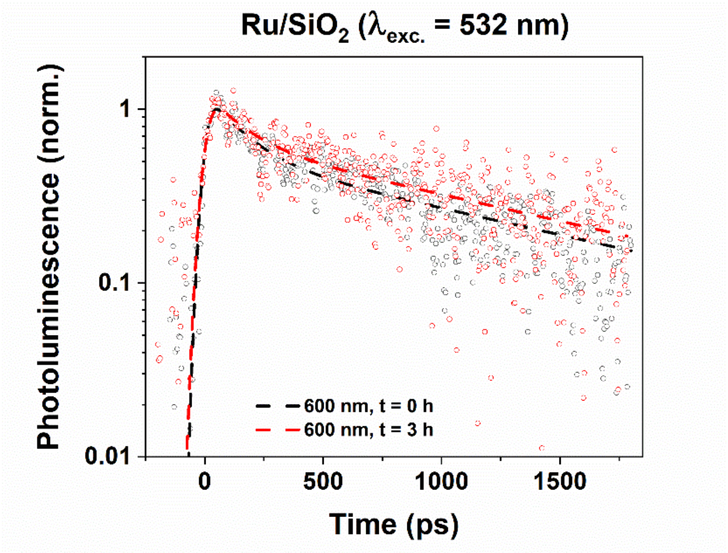

Figure S1. Photoluminescence decays normalized to 1 at 600 nm of Ru/SiO_2 following 532 nm excitation recorded at the beginning of integration and after 3 hours of illumination (described with the same fit based on global analysis using a parallel model with two components, see main text) but with modified amplitude, showing that only the amplitude and not the decay changes in time during integration).

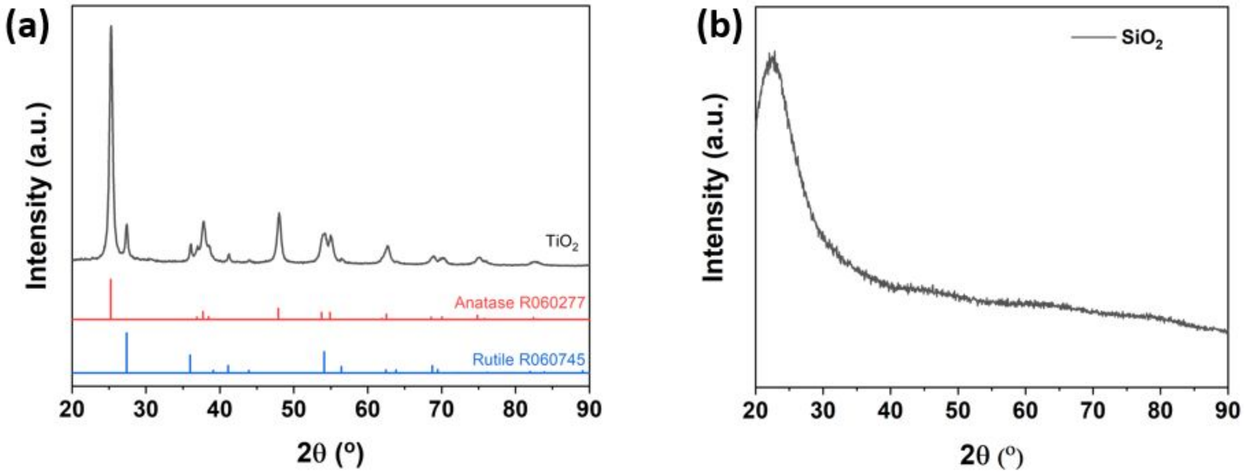

Figure S2. (a) XRD pattern of TiO_2 with reference patterns of anatase and rutile.[1-2] (b) XRD pattern of SiO_2.

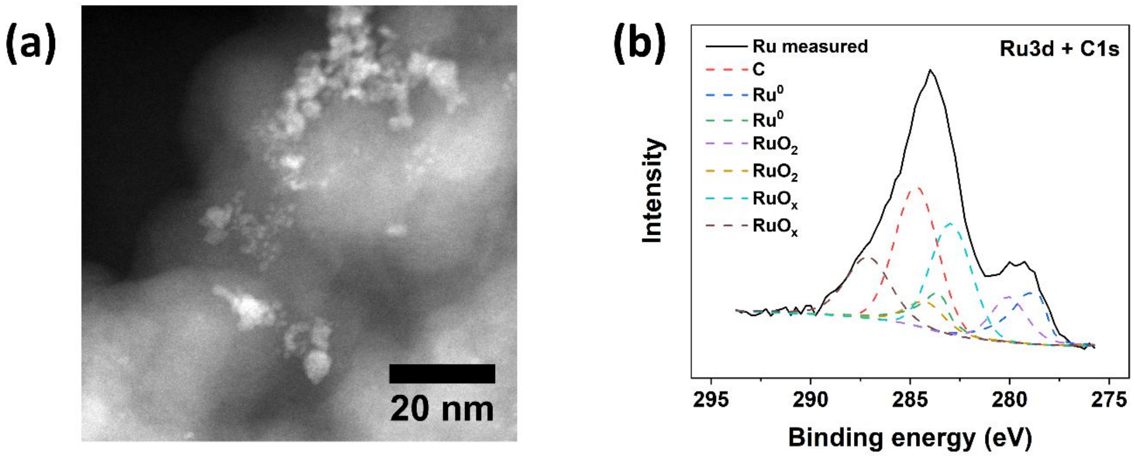

Figure S3. (a) HAADF-STEM image and (b) X-ray photoelectron spectrum and spectral deconvolution of Ru/SiO_2.

Table S1. Atomic percentages of the oxidation states of Ru as detected by XPS.

	**Ru/TiO_2**	**Ru/SiO_2**
**Ru^0**	40.0 %	25.4 %
**RuO_2**	33.5 %	22.1 %
**RuO_x**[a]	26.5 %	52.5 %

a) Ru in RuO_x has a higher oxidation number than Ru in RuO_2, i.e. 5-8.

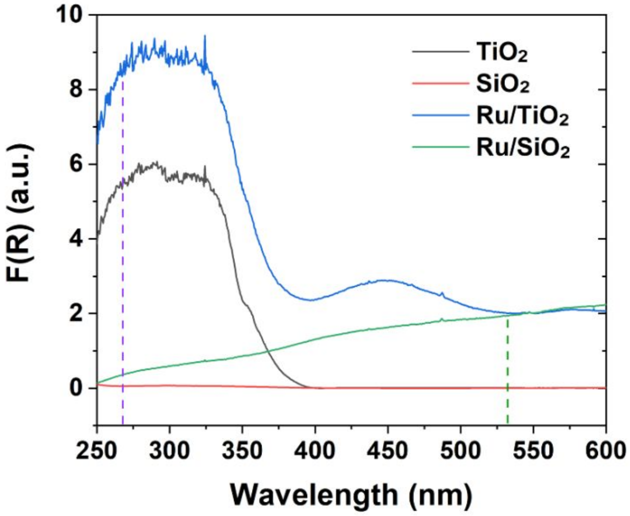

Figure S4. Kubelka-Munk plots of TiO_2, SiO_2, Ru/TiO_2 and Ru/SiO_2.

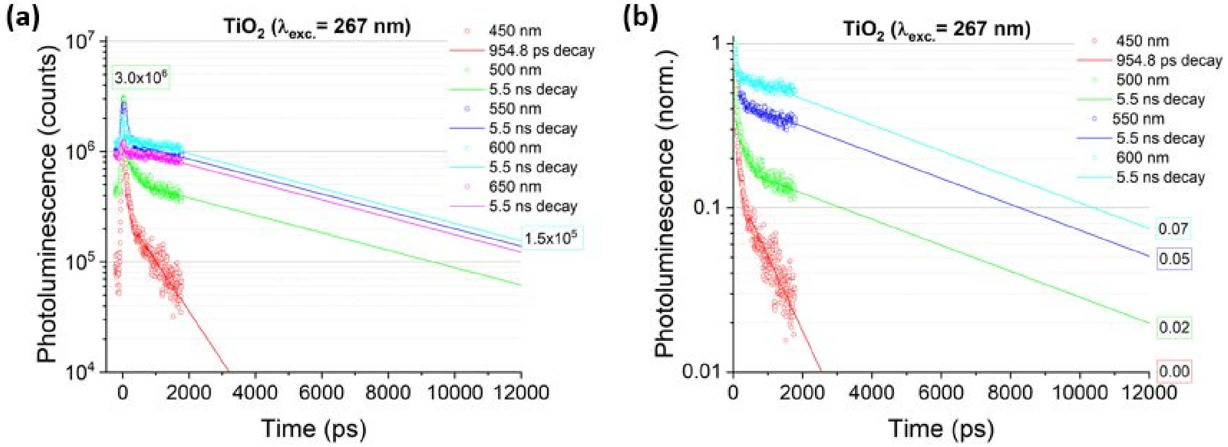

Figure S5. PL decays at the indicated wavelengths of TiO_2 following 267 nm excitation; (a) the absolute intensities and (b) the intensities normalized to 1. The open circles present raw data, the solid lines extrapolated data to 12 ns using the indicated lifetimes. The long-term decay at 450 nm is simulated using a 954.8 ps lifetime (the contribution of DAS3 to the decay at 450 nm is insignificant, see Fig. 3a). The long-term decay of the other PL wavelengths shown is simulated using a 5.5 ns decay, which describes the data well. As this lifetime exceeds the experimentally measured time range, in the photophysical modeling described in the main text we have set this value to ∞.

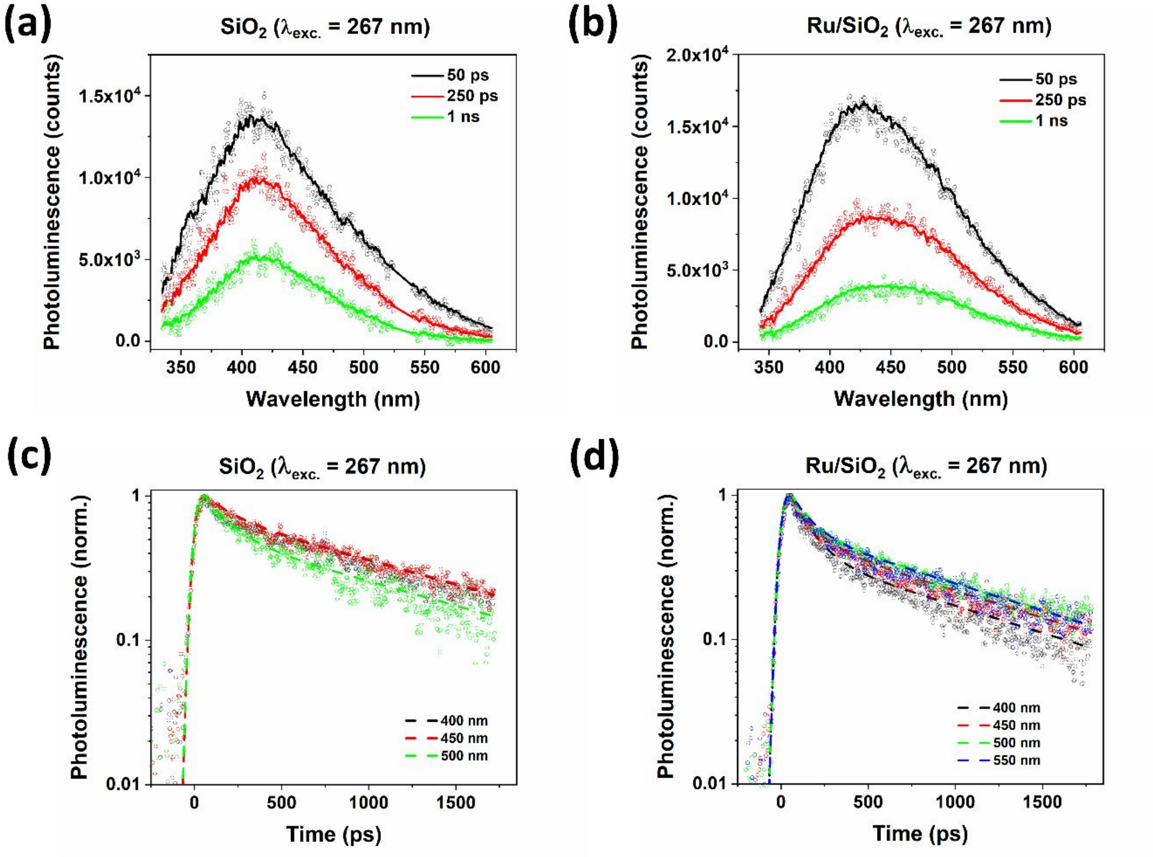

Figure S6. PL spectra at 50 ps, 250 ps and 1 ns after 267 nm 300 fs excitation of nanostructured SiO_2 in Ar (a) and Ru/SiO_2 in Ar (b); (c) and (d) show PL decays normalized to 1 at selected wavelengths. The lines are fits from global analysis using a parallel model with 2 components. For SiO_2, data around 532 nm have been removed because of strong scattering of residual laser light.

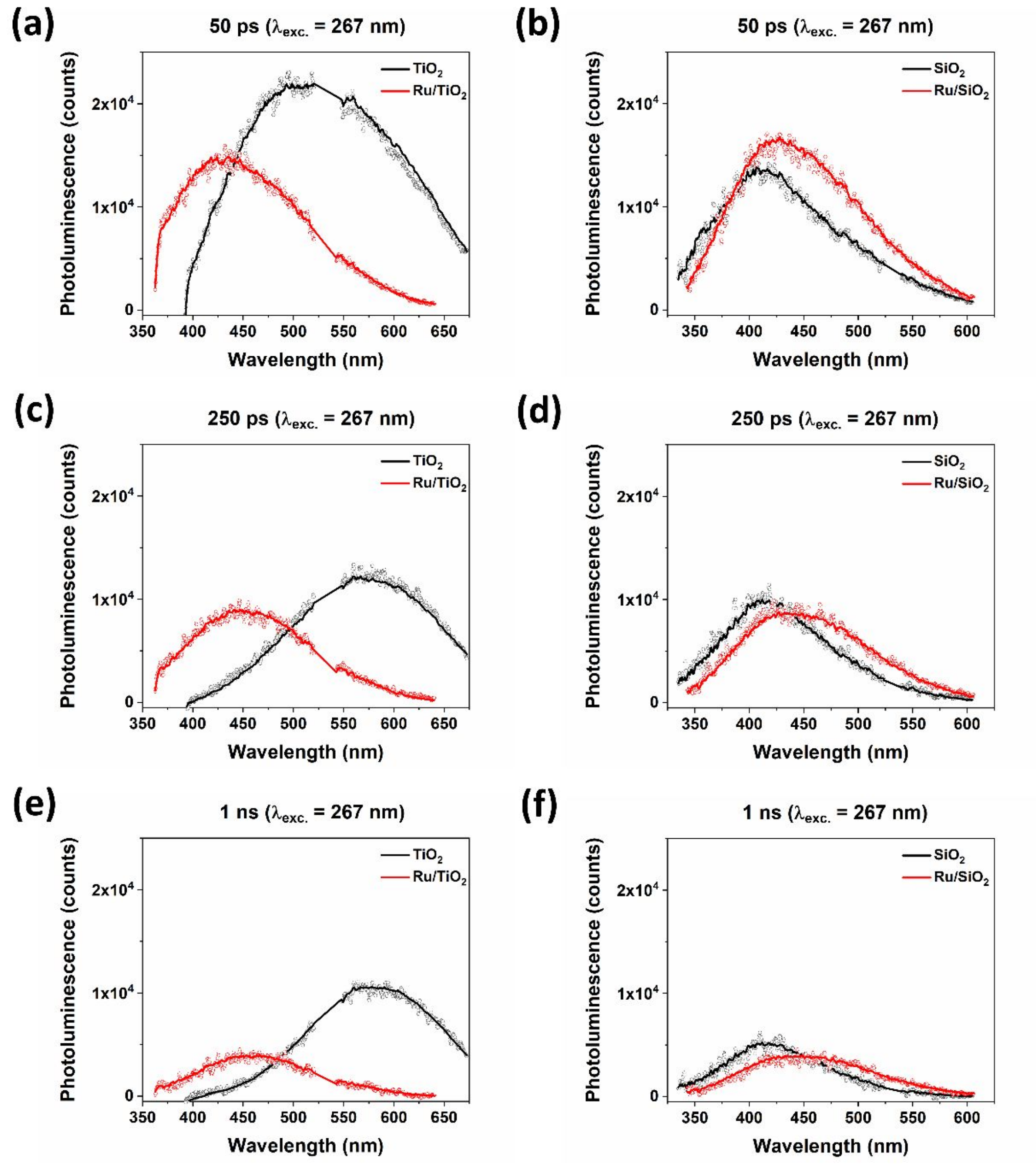

Figure S7. PL spectra at 50 ps (a, b), 250 ps (c, d) and 1 ns (e, f) after 267 nm 300 fs excitation of nanostructured TiO_2 and Ru/TiO_2 in Ar (a, c, e) and nanostructured SiO_2 and Ru/SiO_2 in Ar (b, d,

f). The lines are fits from global analysis using a parallel model with 2 components, with the exception of TiO_2, where a parallel model with 3 components was used. Data around 532 nm have been removed because of scattering of residual laser light, while potential PL<350 nm is blocked by the two 2-inch glass optics used for collecting the PL.

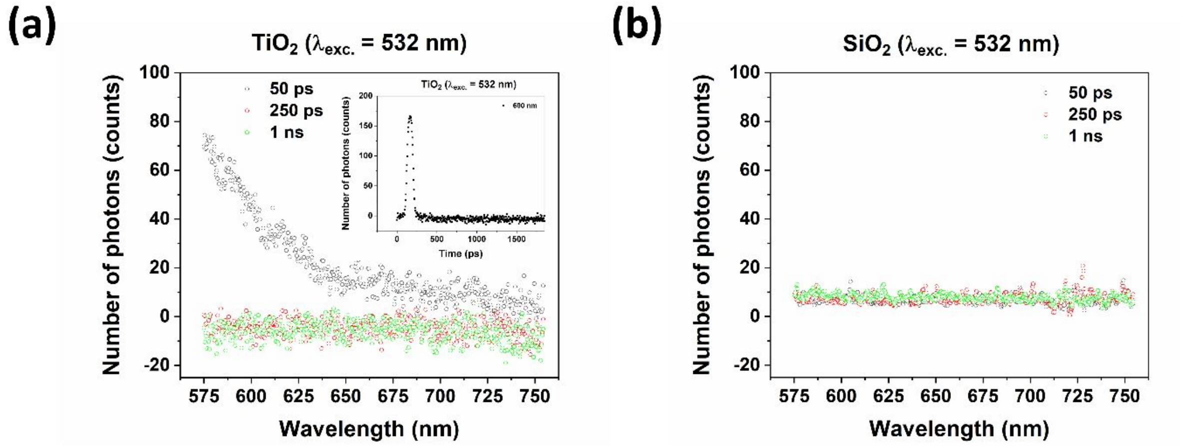

Figure S8. Number of photons as function of wavelength at 50 ps, 250 ps and 1 ns after 532 nm 300 fs excitation of nanostructured TiO_2 (a) and SiO_2 (b). Inset in a: number of photons over time at 600 nm.

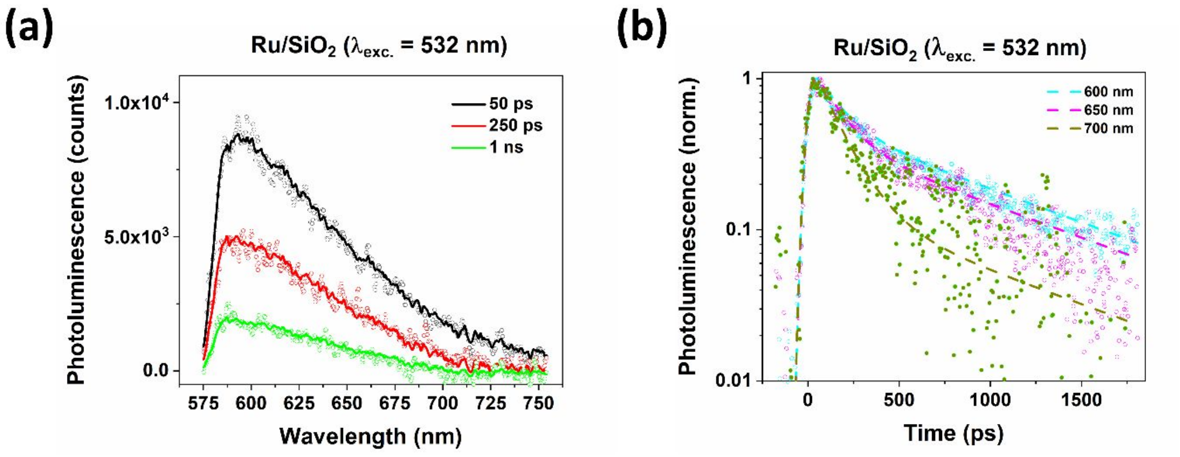

Figure S9. (a) PL spectra at 50 ps, 250 ps and 1 ns after 532 nm 300 fs excitation of nanostructured Ru/SiO_2 in Ar; (b) shows PL decays normalized to 1 at selected wavelengths. The lines are fits from global analysis using a parallel model with 2 components. The PL spectra were recorded using a 570 nm long pass filter to remove residual scattered 532 nm pulses used for photoexcitation.

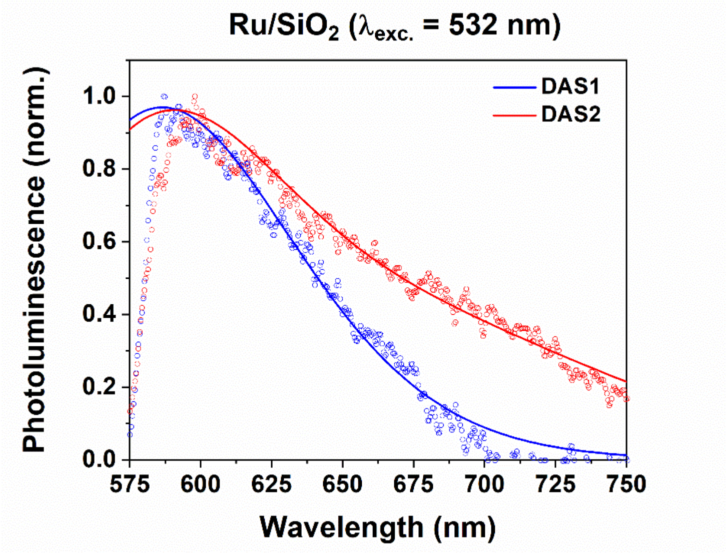

Figure S10. Normalized DAS of Ru/SiO_2 in Ar at 532 nm excitation, recorded through a 570 nm long-pass filter. The solid lines present the sum of Gaussian functions with parameters presented in Table 2 in the main text.

References

1. Horn, M.; Schwerdtfeger, C. F.; Meagher, E. P., Refinement of the structure of anatase at several temperatures. *Zeitschrift für Kristallographie* **1972,** *136* (3-4), 273-281.

2. Meagher, E. P.; Lager, G. A., Polyhedral thermal expansion in the TiO_2 polymorphs: Refinement of the crystal structures of rutile and brookite at high temperature at 425 degrees C. *The Canadian Mineralogist* **1979,** *17*, 77-85.
